# Parameterized absorptive electron scattering factors

**DOI:** 10.1107/S2053273323010963

**Published:** 2024-01-25

**Authors:** M. Thomas, A. Cleverley, R. Beanland

**Affiliations:** a Department of Physics, University of Warwick, Coventry CV4 7AL, UK; b Department of Chemistry, University of Warwick, Coventry CV4 7AL, UK; University of Warsaw, Poland

**Keywords:** electron diffraction, absorption, 3D-ED, three-dimensional electron diffraction, thermal diffuse scattering

## Abstract

In electron diffraction, thermal atomic motion produces incoherent scattering over a relatively wide angular range, which appears as a diffuse background that is usually subtracted from measurements of Bragg spot intensities in structure solution methods. The transfer of electron flux from Bragg spots to diffuse scatter is modelled using complex scattering factors *f* + *if*′ in the Bloch wave methodology. In a two-beam Einstein model the imaginary ‘absorptive’ scattering factor *f*′ can be obtained by the evaluation of an integral containing *f* over all possible scattering angles. While more sophisticated models of diffuse scatter are widely used in the electron microscopy community, it is argued in this paper that this simple model is appropriate for current structure solution and refinement methods. The two-beam model is a straightforward numerical calculation, but even this simplistic approach can become time consuming for simulations of materials with large numbers of atoms in the unit cell and/or many incident beam orientations. Here, a parameterized form of *f*′ is provided for 103 elements as neutral, spherical atoms that reduces calculation time considerably.

## Abstract 2

This paper provides a rapid parameterized calculation of absorptive scattering factors for 103 elements as neutral, spherical atoms, which reduces calculation time considerably.

## 1. Introduction

Crystal structure solution and refinement using electron diffraction has been practised for some time (Vainshtein *et al.*, 1964 ▸) and is currently undergoing a revival. This renewed interest is based on advances in the technique over the past two decades (collectively known as three-dimensional electron diffraction, 3D-ED) (Gemmi *et al.*, 2019 ▸; Gruene *et al.*, 2021 ▸) and the arrival of new dedicated electron diffractometers (Ito *et al.*, 2021 ▸). However, dynamical diffraction effects often limit the accuracy of structures obtained by applying a kinematic scattering model to 3D-ED data, requiring the use of models that take multiple scattering effects into account (Klar *et al.*, 2023 ▸). While most simulations of dynamical electron scattering in electron microscopy are currently performed using the multislice method, the Bloch wave methodology retains some advantages, in particular for continuous-rotation electron diffraction (cRED) data in which low-index zone axes are rare. Bloch wave calculations impose the periodicity of the crystal on the allowed solutions to Schrodinger’s equation but, unlike multislice methods, are not atomistic in their application of boundary conditions, allowing an arbitrary crystal orientation to be simulated without artefacts. In comparison with kinematic intensities calculated from the structure-factor equation, dynamical calculations of intensity are more complex and time consuming, which is a serious concern for iterative refinement of crystal structure. Furthermore, a consequence of only including Bloch waves in a simulation is that only Bragg scattering is accounted for. The inclusion of non-Bragg scattering adds another time penalty to the calculation, which can become appreciable for unit cells containing many atoms.

Here, we provide a rapid parameterized calculation of absorptive scattering factors that eliminates this additional penalty. Current methods do not generally include absorptive scattering factors; their implementation may allow better *R* factors to be obtained and more accurate determinations of crystal structure in future 3D-ED methods.

Strong non-Bragg scattering, in the form of diffuse scatter in electron diffraction patterns, was noticed from the earliest days of electron diffraction (Kikuchi, 1928 ▸; Beeching, 1936 ▸) and was soon identified as being due to inelastic scattering from two principal sources: (*a*) displacement of atoms from their nominal sites due to thermal vibrations (thermal diffuse scattering, TDS), *i.e.* electron–phonon scattering, in which the energy loss is low; and (*b*) other inelastic scattering, principally due to the excitation of plasmons, *Bremsstrahlung*, or ionization of the material’s constituent atoms (a much greater energy loss than for TDS, typically larger than 1 eV). By the 1960s its influence both on diffraction and transmission electron microscopy (TEM) images began to be quantified (Hirsch *et al.*, 1966 ▸), under the term ‘anomalous absorption’ by analogy with the Borrmann effect seen in X-ray scattering (Borrmann, 1941 ▸; Authier & Klapper, 2007 ▸). This term now seems dated but follows from two characteristics – first, while high-energy electrons are not actually absorbed by a thin crystal, the ‘absorption’ of electrons into a diffuse background and consequent attenuation of Bragg scattering follows a similar law to true absorption, and second, the effect is dependent on many parameters, including crystal orientation, scattering vector and incident beam energy.

The first good description of TDS (Hall & Hirsch, 1965 ▸) used a simple model, *i.e.* a crystal with spherical atoms of only one type and one atom per lattice point, which vibrate harmonically and independently (the Einstein model). Using the fact that electrons propagate in a crystal as Bloch waves rather than plane waves, for a given scattering vector **s** they calculated the difference between total elastic scattering 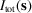 (*i.e.* including the diffuse component) and Bragg scattering 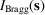 for a two-beam condition. The total non-Bragg scattering is then given by the difference, integrated over the Ewald sphere defining all possible scattering vectors **s**. This framing allows, in the Bloch wave formalism, the transfer of intensity to be dealt with using a complex electron scattering factor of the form *f*_*g*_ + *if*_*g*_′, where *f*_*g*_ is the usual Born electron scattering factor for a diffraction vector with magnitude *g* and *f*_*g*_′ is an imaginary component that depends upon the isotropic Debye–Waller factor *B*_iso_ (Humphreys & Hirsch, 1968 ▸; Hirsch *et al.*, 1966 ▸; Peng, 1997 ▸, 1999 ▸). This complex scattering factor is then used in combination with the temperature factor, 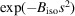 ; for example the structure factor for a reflection **g** is 

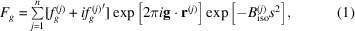

where *s* = sin(θ_B_)/λ = *g*/2 and the summation is performed over all *n* atoms in the unit cell. It is found that *f*′ decays more rapidly with angle, and is typically an order of magnitude smaller than *f*. Due to the computational cost of the Hall and Hirsch approach, most working calculations at the time instead used a proportional model in which *f*_*g*_′ = α*f*_*g*_, typically with α ∼ 0.1 (Humphreys & Hirsch, 1968 ▸). Subsequent work expanded the approach to include core-loss scattering (Radi, 1970 ▸; Rossouw & Bursill, 1985*b* ▸, 1986 ▸; Rossouw, 1985 ▸; Allen & Rossouw, 1990 ▸). Rossouw also extended the TDS model to a full dynamical *n*-beam case for both the incident and scattered waves (Rossouw & Bursill, 1985*a* ▸,*b* ▸). In the context of microscopy, TDS has received a great deal of attention as the primary signal in atomic resolution scanning transmission electron microscopy (STEM) (*e.g.* Pennycook & Jesson, 1991 ▸; Rossouw *et al.*, 2003 ▸; Croitoru *et al.*, 2006 ▸; Klenov & Stemmer, 2006 ▸; Rosenauer *et al.*, 2008 ▸).

The sophistication of the latter models of diffuse electron scattering is essential for a complete description of a diffraction pattern, particularly when the crystal is aligned to a low-index zone axis with many beams excited simultaneously. This comes at a computational cost – Rossouw’s *n*-beam dynamical calculations of TDS scale as *n* ^8^ – which is impractical to include in iterations of models when refining a crystal structure obtained from cRED data. It is therefore important to find the best compromise, *i.e.* use a model of sufficient accuracy for the technique of interest whilst minimizing the computational overhead. In the measurement of diffracted intensities, describing diffuse scatter as ‘absorption’ equates to an assumption that these electrons do not return into Bragg scattered spots (or do so in a way that can readily be subtracted). For such measurements, an important distinction should be made between TDS and higher energy-loss inelastic scattering, since the latter is strong only at very small scattering angles, while TDS produces a broad diffuse background across the whole pattern. Thus, inelastic scattering effectively acts to blur a diffracted spot, or dynamical features in convergent-beam patterns (Tanaka *et al.*, 2002 ▸) and will still be included in a measurement of diffracted intensity. Conversely, the broad TDS background is usually subtracted from a measurement (Palatinus *et al.*, 2019 ▸) and therefore considering it to be ‘absorption’ is an appropriate model. For structure solution using cRED, where low-index zone axes are encountered infrequently and absorption is already a second-order effect, a simple model will suffice. While Rossouw did not quantify the difference between a two-beam model and an *n*-beam model, the effect is principally to change the distribution of diffuse intensity at zone axes where multiple channelling pathways exist (Rossouw & Bursill, 1985*b* ▸). Furthermore, as shown by Peng (1997 ▸), anisotropic thermal vibrations produce similar changes in both real and imaginary parts of the complex crystal potential, indicating that it should generally be acceptable to simply replace *f*_*g*_ with *f*_*g*_ + *if*_*g*_′ in an electron diffraction refinement.

## 2. Calculation

Calculations of absorptive TDS scattering factors *f*_*g*_′ based on the two-beam model were given by Bird & King (1990 ▸) and Weickenmeier & Kohl (1991 ▸), who provided some tabulated values and computer code allowing their calculation. While some tabulated values were given for a limited set of elements and compounds for 100 kV electrons by Peng *et al.* (1996*a* ▸,*b* ▸), no general parameterized version exists that would enable a rapid calculation for all elements, which is our purpose here. We use the calculation of Bird & King (1990 ▸), who employed an elegant change of variable to give 

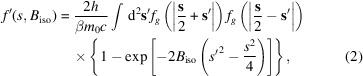

integrated over the Ewald sphere for *s*′, where *h* is Planck’s constant, *m*_0_ the rest mass of the electron, *c* the speed of light and β the velocity ratio *v*/*c*. Usefully, this framing of the problem gives a fourfold symmetry of the integrand, allowing numerical integration to be performed over 0 ≤ *s*′ < ∞ in two dimensions which avoids most issues with poor convergence.

We evaluate the integral of equation (2), moving the factor β to give a result that is independent of accelerating voltage, using the Born electron scattering factors of Lobato & Van Dyck (2014 ▸) for neutral atoms. For each element we give a parameterized β*f*′(*s*, *B*_iso_) that is a sum of four Gaussian functions, each of which is determined by two parameters *a*_*i*_, *b*_*i*_. This is in addition to a single constant term *c*, giving a total of nine parameters tabulated for each value of *B*_iso_, *i.e.*


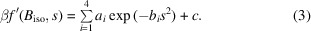




A non-linear least-squares fit of equation (3) to equation (2), evaluated over 100 values of *s* (0 ≤ *s* ≤ 6 Å^−1^), for 103 elements was performed using a Trust Region reflective algorithm (Branch *et al.*, 1999 ▸), providing a parameterized approximation of *f*′ for a given value of *B*_iso_ (0.1 ≤ *B*_iso_ ≤ 4 Å^2^). This approach is more straightforward in use than the *f*′/*f* over a grid of *B*_iso_ *s* ^2^ and *B*_iso_ given by Bird & King (1990 ▸). In equation (1), multiplying by the temperature factor, 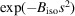 , ensures that the function smoothly asymptotes to zero. Without it, the absorptive form factor instead behaves as 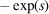 for large *s* paired with any *B*_iso_ > 0.05 Å^2^ (Peng *et al.*, 1996*b* ▸). This results in large negative β*f*′ at large *s*, which implies amplification, rather than absorption, of the electron beam and we consider this to be unphysical. We therefore set β*f*′ to zero where equation (2) returns a negative value. Note that equation (1) does not present this behaviour to such an extreme; only when the temperature factor is removed do the unphysical results strongly present themselves. Intermediate values of β*f*′ may be obtained by linear interpolation of the four nearest values, while negative values may be disregarded. In any implementation the accelerating voltage must be taken into account both by including β and multiplying by the relativistic correction γ = 1/(1 − *v* ^2^/*c* ^2^)^1/2^, to give the complex scattering factor γ*f* + *i*γ*f*′.

## 3. Results

**Figure 1:**
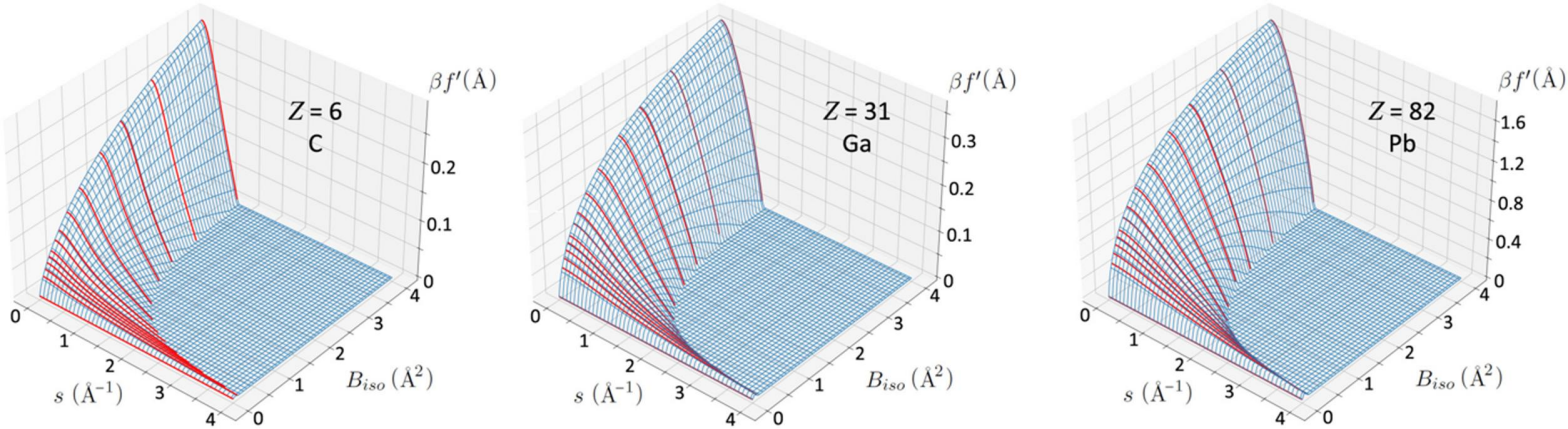
Plot of β*f*′ for carbon (*Z* = 6), gallium (*Z* = 31) and lead (*Z* = 82) over the range 0 < *s* < 4 and 0 < *B*_iso_ < 4 calculated from equation (2) (blue) and parameterized at 13 values of *B*_iso_ (red).

**Figure 2:**
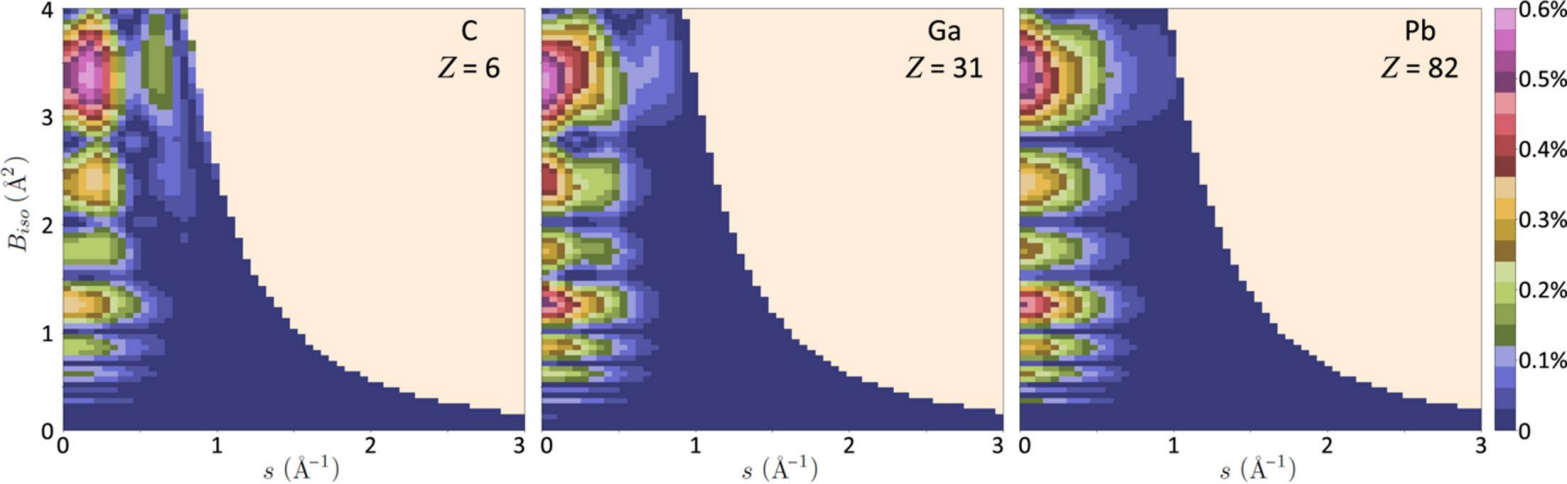
Errors resulting from linear interpolation between the parameterized values of 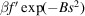 , compared with those calculated from the integral of equation (2) for carbon (*Z* = 6), gallium (*Z* = 31) and lead (*Z* = 82). The range of scattering vector [*s* = sin(θ)/λ = *g*/2] is 0 < *s* < 3 and isotropic Debye–Waller factor is 0 < *B*_iso_ < 4. Values are given as fractions of the maximum value of 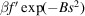 over the mapped range. In the blank region, *f*′ = 0.

Fig. 1 ▸ shows the absorptive electron scattering factor over a range of *B*_iso_ and *s* for selected light, intermediate and heavy atoms, *i.e.* C, Ga and Pb, respectively (note different scales; like normal scattering factors, absorptive scattering factors increase with atomic number). The maximum value of *f*′ at *s* = 0 increases rapidly with *B*_iso_, but rapidly drops to zero around *s* ∼ 1 Å^−1^ at high temperatures (large *B*_iso_). Conversely, below *B*_iso_ ∼ 0.5 Å^2^ it extends to much larger *s*. Thirteen values of *B*_iso_ were chosen to be parameterized, as a compromise between the best accuracy and a compact calculation that does not require a large number of parameters (here, 9 × 13 = 117) for each element. In general, the parameterized value of *f*′ is well within 0.1% of that resulting from equation (2) and the curves are indistinguishable in Fig. 1 ▸. However, the curvature of the surfaces in Fig. 1 ▸ causes the parameterized *f*′, which is obtained using a simple linear interpolation, to be slightly less reliable between the chosen values of *B*_iso_. The extent of these errors is shown in Fig. 2 ▸, which gives a map of the difference between parameterized and directly calculated absorptive scattering factors. To avoid artefacts these maps include the temperature factor 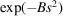 ; they are otherwise dominated by errors in very small values of *f*′ at the boundary where the curve passes through zero, which occur due to evaluation over a finite grid. These errors are negligible in practice since they are suppressed by the factor 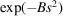 . In order to allow comparison between different elements, the error is normalized by the maximum value of 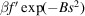 in the grid. While this means that the absolute values are somewhat arbitrary, it shows that they remain very small fractions of the calculated β*f*′.

**Figure 3:**
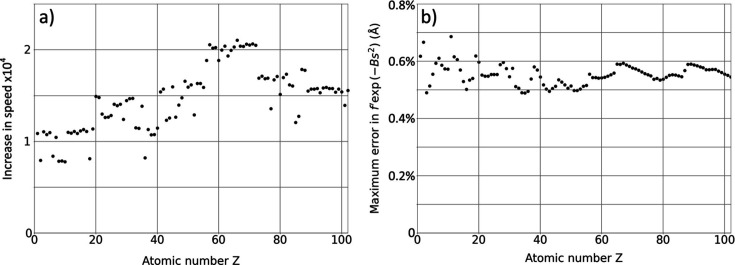
(*a*) Increase in speed of calculation as measured on a Windows 11 64 bit desktop. Variations are mainly due to competing windows processes but generally exceed 10 000× with a slight improvement at higher atomic numbers. (*b*) The maximum error in 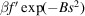 resulting from linear interpolation between the parameterized values at fixed *B*_iso_, evaluated in the same way as in Fig. 2 ▸.

Measurements of the speed improvement derived from parameterized *f*′ in comparison with the direct calculation are given in Fig. 3 ▸(*a*), evaluated for 1000 function calls to a Python script on a Windows 11 machine. Parameterized *f*′ were typically returned in 30 µs, while the direct calculation required between 300 and 600 ms. While significant variability is present, a small improvement with increasing atomic number is apparent. Fig. 3 ▸(*b*) gives an evaluation of errors using the same method as in Fig. 2 ▸. The lack of dependence on atomic number indicates that the method gives good results, which should be generally applicable to all elements and compounds. A Python subroutine that returns the complex scattering factor γ*f* + *i*γ*f*′ for input *B*_iso_, *s* and accelerating voltage *V* is provided in the supporting information and is also available online (Thomas, 2023 ▸).

## 4. Discussion and conclusions

In summary, we have parameterized absorptive scattering factors using the method described by Bird & King (1990 ▸). The impact of ‘absorption’ into a diffuse background on the measurement of diffracted intensities, used for structure solution with electron diffraction, is currently not quantified. It is, however, clear that crystal structure refinements based on dynamical simulations show significant improvements over kinematical ones (Klar *et al.*, 2023 ▸; Cleverley & Beanland, 2023 ▸) and it is probable that, with sufficiently accurate dynamical simulations and high-quality data, these absorption effects will become evident to a similar extent to that seen in the more established methods of CBED (convergent-beam electron diffraction), TEM and STEM. The method applied here neglects diffuse scattering due to higher-energy (plasmon and core-loss) inelastic scattering on the grounds that it is generally limited to relatively small angles in comparison with TDS. This assumption will inevitably hold less well for thicker crystals, for which the TDS also becomes inelastically scattered through these mechanisms. As shown by Yang *et al.* (2022 ▸), the removal of inelastic scattering by energy filtering can give significant improvements in quality-of-fit indices such as *R*_1_, although the details of diffuse background subtraction in their data processing (using code developed for X-ray diffraction) and the thickness of the crystals they used were not given. Further work is still required to determine whether the effort needed to quantify, or remove, this additional effect by energy filtering is worthwhile for structure solution and refinement. In addition, it is important to note that multiple scattering of electrons in the diffuse background also takes place, adding structure such as Kikuchi lines and complex variations of intensity at low-index zone axes, which may affect measurements of the intensity of a Bragg peak. It is thus certain that, as precision and accuracy improve in electron diffraction methods, these effects will become more visible.

Anisotropic thermal vibrations are routinely determined in crystal structure refinements. As a first approach to an anisotropic model in electron diffraction, it may be sufficient to simply use the complex structure factor *f* + *if*′ in place of *f*. However, a more appropriate method to obtain an anisotropic form of *f*′ would be to reframe the integral of equation (2) using the anisotropic tensor form of *f*, which will give a slightly different result. A further important aspect is the effect of charge transfer, ionicity and multipolar atomic models. The change in structure factor can be significant at low *s* and there is already clear evidence that it is readily detectable in cRED electron diffraction data (Gruza *et al.*, 2020 ▸). Inclusion of these effects in the simple absorption model used here can simply be achieved by use of the appropriate Born scattering factor *f* in equation (2). However, like the other more sophisticated models mentioned above, it remains to be determined whether the additional computational cost is worthwhile in structure solution and refinement using electron diffraction.

While evaluation of the integral of equation (2) is not difficult using modern numerical methods, it remains relatively slow and could limit simulations of materials with large unit cells and many atoms. It is hoped that the parameterized versions provided here will allow the inclusion of absorption in the rapid calculations that will be necessary in routine refinements of crystal structure.

## 5. Software and data availability

All code used in this work is available online (Thomas, 2023 ▸) and on the Warwick Research Archive Portal https://wrap.warwick.ac.uk/181354/.

## Supplementary Material

Python code for returning the complex scattering factor. DOI: 10.1107/S2053273323010963/pl5034sup1.zip
